# Photoplethysmography respiratory rate monitoring in patients receiving procedural sedation and analgesia for upper gastrointestinal endoscopy

**DOI:** 10.1007/s10877-016-9890-0

**Published:** 2016-05-28

**Authors:** Hugo R. W. Touw, Milou H. Verheul, Pieter R. Tuinman, Jeroen Smit, Deirdre Thöne, Patrick Schober, Christa Boer

**Affiliations:** 1 Department of Anaesthesiology, Institute for Cardiovascular Research, VU University Medical Centre, De Boelelaan 1117, 1081 HV Amsterdam, The Netherlands; 2 Department of Intensive Care Medicine, Institute for Cardiovascular Research, VU University Medical Centre, De Boelelaan 1117, 1081 HV Amsterdam, The Netherlands

**Keywords:** Respiratory rate, Sedation, Anoxia, Hypoxaemia, Apnoea, Procedural sedation and analgesia

## Abstract

The value of capnography during procedural sedation and analgesia (PSA) for the detection of hypoxaemia during upper gastrointestinal (UGI) endoscopic procedures is limited. Photoplethysmography respiratory rate (RRp) monitoring may provide a useful alternative, but the level of agreement with capnography during PSA is unknown. We therefore investigated the level of agreement between the RRp and capnography-based RR (RRc) during PSA for UGI endoscopy. This study included patients undergoing PSA for UGI endoscopy procedures. Pulse oximetry (SpO_2_) and RRc were recorded in combination with Nellcor 2.0 (RRp) monitoring (Covidien, USA). Bland–Altman analysis was used to evaluate the level of agreement between RRc and RRp. Episodes of apnoea, defined as no detection of exhaled CO_2_ for minimal 36 s, and hypoxaemia, defined as an SpO_2_ < 92 %, were registered. A total of 1054 min of data from 26 patients were analysed. Bland–Altman analysis between the RRc and RRp revealed a bias of 2.25 ± 5.41 breath rate per minute (brpm), with limits of agreement from −8.35 to 12.84 brpm for an RR ≥ 4 brpm. A total of 67 apnoea events were detected. In 21 % of all apnoea events, the patient became hypoxaemic. Hypoxaemia occurred 42 times with a median length of 34 (19–141) s, and was preceded in 34 % of the cases by apnoea and in 64 % by an RRc ≥ 8 brpm. In 81 % of all apnoea events, photoplethysmography registered an RRp ≥ 4 brpm. We found a low level of agreement between capnography and the plethysmography respiratory rate during procedural sedation for UGI endoscopy. Moreover, respiratory rate derived from both the capnogram and photoplethysmogram showed a limited ability to provide warning signs for a hypoxaemic event during the sedation procedure.

## Introduction

Upper gastrointestinal (UGI) endoscopy is frequently performed under procedural sedation and analgesia (PSA) to facilitate the procedure and enhance patient comfort. However, PSA might be associated with intraprocedural sedation-related cardiovascular and respiratory events, such as depressed minute ventilation and eventually hypoxaemia defined as an SpO_2_ < 92 %, especially in patients with a higher ASA classification and body mass index [1, 2]. Pulse oximetry is the most commonly used monitoring parameter for the detection of hypoxaemic episodes during PSA, but it is a late indicator of hypoventilation, especially in patients that receive supplemental oxygen [3–5]. Alternatively, it has been suggested that capnography during PSA may identify respiratory depression before the onset of hypoxaemia [6–8]. However, while capnography is currently considered the gold standard of respiratory rate monitoring in perioperative medicine, its usefulness during PSA for UGI is under debate. Although capnography might reduce hypoxaemic events by early detection of ventilation abnormalities [8], Quadeer et al. [8] also reported that capnography erroneously displayed a flat line for at least 50 s without a concomitant decrease in oxygen saturation with normal chest excursions on subsequent clinical examination in 13 % of patients during PSA for UGI endoscopy. Concordantly, 25 % of patients who develop hypoxaemia show normal ventilation patterns according to capnography [9], and 46 % of patients with capnography monitoring developed hypoxaemia [8]. This inaccuracy is mainly caused by a diminution of airflow due to the presence of the oral scope in a narrow oropharyngeal inlet, interference of the CO_2_ suction pump with capnographic CO_2_ detection, or blockage of the nasal cannula by residual moisture accumulation [8].

Respiratory rate is considered as a proxy of minute ventilation [10, 11], and the introduction of monitors that enable continuous registration of respiration rate, including transthoracic impedance plethysmography or bioacoustical sensor technology, may broaden the monitoring spectrum during PSA. The recently introduced Nellcor™ photoplethysmography (PPG) waveform analysis calculates the respiratory rate from the respiratory variation in the pulse oximetry signal [12–14]. The added value of the use of PPG-based respiratory rate (RRp) monitoring technology during PSA has however not yet been evaluated. In this study we therefore investigated the level of agreement between respiratory rate monitoring based on photoplethysmography or capnography during PSA for UGI endoscopic procedures and evaluated the ability of RRp to predict hypoxaemia in this setting.

## Methods

### Subjects

This prospective, observational clinical study was conducted in the VU University Medical Centre (Amsterdam, The Netherlands), a tertiary hospital. The local Human Subjects Committee of the VUmc approved the study (METc14/489), and written informed consent was obtained from all subjects. Patients were included in case of procedural sedation and analgesia (PSA) for upper gastrointestinal (UGI) endoscopy. Patients were excluded from enrolment if they met any of the following exclusion criteria: <18 years of age, mechanical ventilation, atrial fibrillation and the presence of an implanted pacemaker.

### PSA

PSA was conducted according to the national guideline and local protocols for the administration of PSA outside the operating room. Propofol was administered via a target-controlled infusion (TCI) system (120–185 µg kg^−1^ min^−1^). The dosage of propofol, (s)-ketamine (Ketanest, Pfizer, the Netherlands) and alfentanil (Rapifen, Janssen-Cilag BV, the Netherlands) was individualized and titrated to the desired clinical effect by a certified registered nurse anaesthetist specialized in PSA with a supervising anaesthetist available for assistance. Standard in all procedures 3 L/min of supplemental oxygen was administered via a nasal cannula.

### Standard respiratory monitoring

Pulse oximetry (SpO_2_), heart rate (HR) and intermittent non-invasive blood pressure (NIBP) were monitored with an IntelliVue MX450 and capnography with an M3015B module (Koninklijke Philips NV^®^, Eindhoven, the Netherlands). A sidestream and/or microstream^®^ CO_2_ filter Smart CapnoLine Guardian™ (Covidien, Mansfield, MA, USA) was used for measuring CO_2_ via a combined nasal and oral cannula. The IntelliVue system displays respiratory rate (RRc), end-tidal CO_2_ levels and a continuous capnographic waveform during the UGI endoscopic procedure. The RRc that was recorded consists of the average breath rate per minute (brpm) calculated every 12 s, and is able to reflect an RR from 0 to 40 brpm. The capnometer was set to alarm when there was no CO_2_-detection.

### Plethysmography-based respiration rate monitoring

The Nellcor™ bedside patient monitoring system version 2.0 (Covidien, Mansfield, MA, USA) was used to measure the respiratory rate derived from the photoplethysmogram (RRp). The finger cuff of the Nellcor was wrapped around the finger and placed ipsilateral to the NIBP. The hand was subsequently placed in a supine position. Every 5 s, the Nellcor calculates a current respiratory rate based on a 45-s photoplethysmogram. This RRp is averaged further with the previously displayed rate, and passes through additional logic before displayed as a final RRp reported to the user. The Nellcor has a reference range of 4–40 brpm and therefore suppresses the display of respiratory rates <4 brpm. The entire algorithm is reset (returns to NO POST) if the oximeter algorithm reports a pulse rate or SpO_2_ of zero (i.e. a dropout) or the sensor was disconnected [15]. No alarms were set on the Nellcor device.

### Data extraction

RRc and RRp measurements were performed simultaneously in sedated patients who underwent UGI endoscopy. Data recording started when medication was administered. For every patient, respiratory rates were converted into a mean RR per minute for both capnography (average of 5 measurements) and photoplethysmography (average of 60 measurements). The two parameters were matched exactly with respect to the time point and time frame. SpO_2_ and heart rate data were recorded every second in the offline IntelliVue data report. NIBP values were collected every 5 min.

A hypoxaemic episode was defined as any timeframe with an SpO_2_ value below 92 %. Episodes of hypoxaemia were counted and the average length was calculated. The episodes of hypoxaemia were categorized according to the registered RR prior to hypoxaemia: non-registration, normopnoea, bradypnoea and apnoea. Bradypnoea was defined as an RRc of 1–7 brpm or RRp of 4–7 brpm. The number and average length of detected bradypnoea episodes was calculated for both RRc and RRp. When a hypoxaemic episode was preceded by bradypnoea, the elapsed time between the occurrence of bradypnoea and the signalling of a SpO_2_ below 92 % was calculated for both monitors.

Episodes of apnoea captured by capnography were defined as a time lapse exceeding 36 s, or no detection of exhaled CO_2_. The performance quality of monitoring was registered by calculating the percentage and average duration of non-registration during the procedure, and defined as the inability to calculate the respiratory rate per minute.

### Other study procedures

All interventions performed by the sedation nurse when inadequate ventilation or oxygenation was recognized were recorded. These interventions included basic life support, airway manoeuvres (e.g. jaw-thrust, head-tilt and chin-lift) and ventilator support with a manual resuscitator.

Other study parameters, patient characteristics, comorbidities, medical history, ASA classification and type of procedure were recorded on a case record form. Relevant changes in blood pressure, heart rate, hand movement and surrounding noise where marked as an event on the Nellcor monitor. After removal of the endoscopic device, the total amount of administered propofol, s-ketamine and alfentanil were recorded.

### Statistical analysis

Statistical data analyses were carried out using a SPSS statistical software package version 19.0 (IBM, New York, NY, USA). Standard descriptive statistics were used to describe the patient characteristics and respiratory data and expressed as mean ± standard deviation (SD), median with interquartile range (IQR) or frequencies.

The bias and limits of agreement (LoA) between the RRc and RRp were analysed using Bland–Altman analysis for repeated measurements using MedCalc version 12.7.4. (MedCalc Software, Ostend, Belgium). Bland–Altman analysis provided the bias, SD of the bias, and limits of agreement between both methods for an RR ≥ 4 brpm. The 95 % limits of agreement refer to the bias ±1.96 SD.

Furthermore, the relation between the RRc and the RRp was evaluated by linear regression analysis using (RRp + RRc)/2 as the independent variable and RRp–RRc as the dependent variable. Regression analysis was used to quantify whether the difference between measurements for both monitors was related to the average measured RR. This analysis was not corrected for multiple measurements.

Additionally, Bland–Altman analyses were performed in case of an RR ≥ 12 brpm, which is considered as the lower limit of a normal breathing frequency. A statistical significant difference was defined as a *p* value <0.05.

## Results

### Patient characteristics

**Fig. 1:**
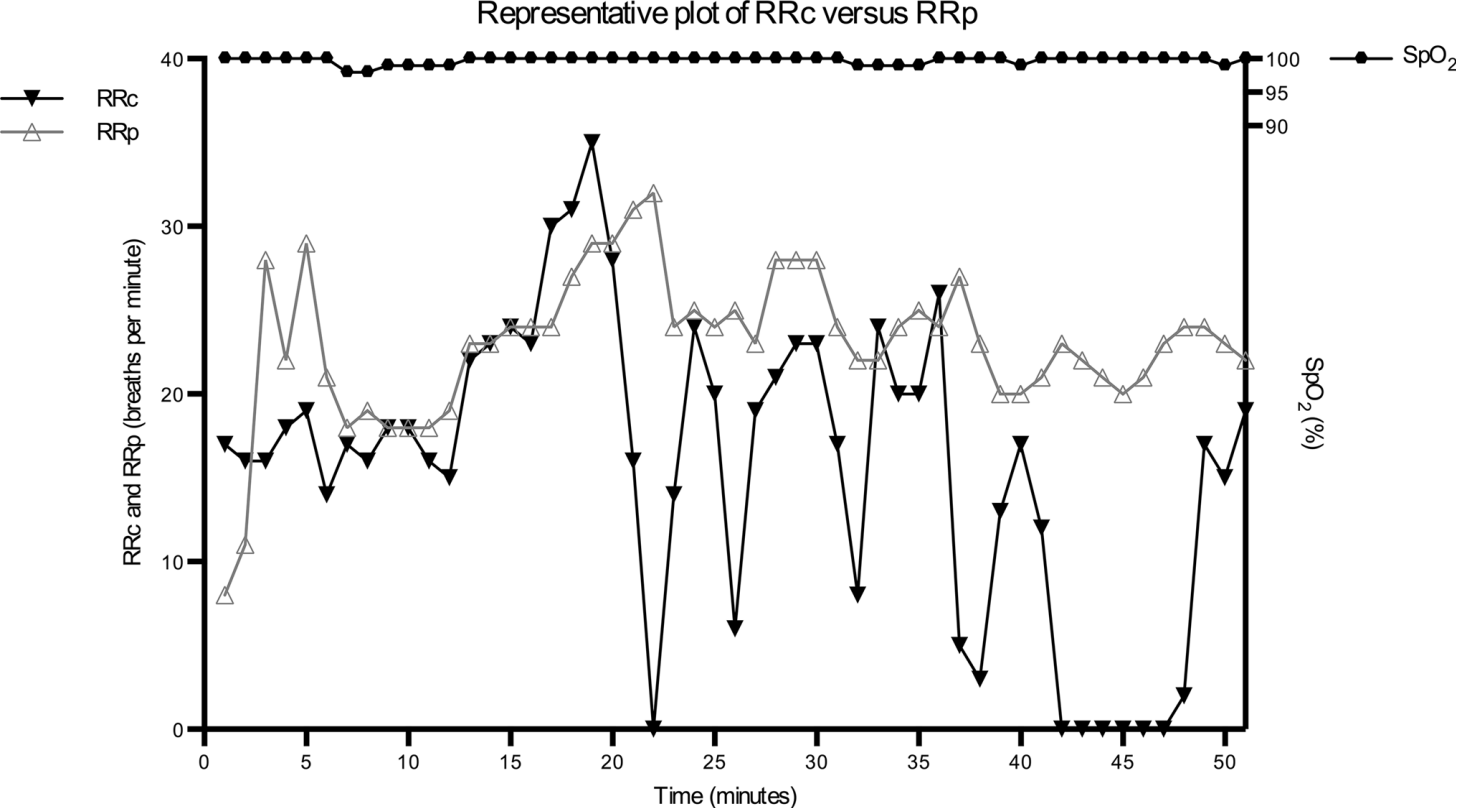
Capnography, photoplethysmography and pulse oximetry data of a representative patient receiving PSA during UGI endoscopy

Twenty-eight patients were assessed for eligibility and included in the study. One patient was excluded from the final analysis due to the development of atrial fibrillation de novo during the procedure. A second patient was excluded, as pulse oximetry data could not consistently be recorded. A total of 1054 min of capnography and photoplethysmography data points were obtained. Figure 1 shows the capnography, photoplethysmography and pulse oximetry data of a representative patient receiving PSA during UGI endoscopy. Table 1 shows the characteristics of the included patients. The patient population consisted of 16 males and 10 females, they were 59 ± 16 years old and had a median ASA classification of 2 (1–3).

**Table 1:** Characteristics of the study population

Patient characteristics	Values
N	26
Males/females	16/10
Age (years)	59 ± 16
Body mass index (kg/m^2^)	23.1 ± 4.8
ASA score	2 (1–3)
Alcohol use [n (%)]	8 (31)
History of smoking [n (%)]	9 (35)
Comorbidities [n (%)]	
Chronic obstructive pulmonary disease	3 (12)
Obstructive sleep apnoea	1 (4)
Hypertension	8 (31)
Cardiomyopathy	1 (4)
Heart failure	1 (4)
Renal failure	2 (8)
Cirrhosis	1 (4)
Diabetes mellitus	3 (12)

Data represent mean ± SD, median with interquartile range or frequencies

*ASA* American Society of Anaesthesiologists

### Procedural characteristics

The characteristics of the procedural sedation and anaesthesia are detailed in Table 2. Most patients underwent oral double balloon enteroscopy (35 %) or endoscopic retrograde cholangiopancreatography (31 %). The median procedural duration was 36 (25–64) minutes. The average respiratory rate during the procedure was 12 ± 8 breath rate per minute (brpm) with a SpO_2_ of 97 ± 3 %. Propofol was administered in combination with alfentanil (85 % of the patients) and/or S-ketamine (77 % of the patients). The RRc and RRp ranged from 0 to 36 and 4 to 32 brpm, respectively.

**Table 2:** Upper gastrointestinal endoscopy procedural characteristics

Procedural and patient characteristics	Values
Type of procedure [n (%)]	
Oral double balloon enteroscopy	9 (35)
Endoscopic retrograde cholangiopancreatography	8 (31)
Gastroscopy	5 (19)
Oesophageal dilatation	1 (4)
Upper endoscopic ultrasound	1 (4)
Percutaneous endoscopic gastrostomy	1 (4)
Procedural duration (min)	36 (25–64)
Manual resuscitation [n (%)]	1 (4)
Median heart rate (bpm)	77 (71–97)
Mean respiratory rate (brpm)	12 ± 8
Mean SpO_2_ during procedure (%)	97 ± 3
TCI propofol (mg)	365 (245–521)
TCI propofol (mg kg^−1^ h^−1^)	8.3 (7.1–10.5)
Alfentanil use (μg)	196 (100–300)
S-ketamine use (mg)	15 (0–25)

Data represent mean ± SD, median with interquartile range or frequencies

*Bpm* beats per minute, *brpm* breaths per minute, *TCI* target controlled infusion

### Episodes of hypoxaemia and apnoea

Hypoxaemia was detected 42 times in 11 patients (39 %), with a median episode length of 34 (19–141)s. Thirty-seven out of 42 (88 %) hypoxaemic episodes occurred in the first 10 min of the sedation procedure.

In 14 (34 %) cases, the hypoxaemic episode was preceded by apnoeas detected by capnography, with a delay of 40 s between apnoea and the development of hypoxaemia. In 10 cases (24 % of all hypoxic events), the RRp could not be measured or display of the RRp was suppressed by the Nellcor algorithm in the period preceding a hypoxaemic period. Bradypnoea before a hypoxaemic episode was detected in 1 and 2 cases by capnography and photoplethysmography, respectively. A respiratory rate ≥8 brpm preceded a hypoxaemic episode in 64 % (RRc) and 71 % (RRp) of the cases. Table 3 shows the characteristics of episodes of apnoea as detected by capnography. A total of 67 apnoeas were detected by capnography with in incidence of 36 % for the total patient cohort and a median length of 159 (68–198) seconds.

**Table 3:** Characteristics of apnoea detection by capnography

Apnoea episodes	Values
Total of detected episodes of apnoea	67
Episode length(s)	159 (68–198)
Number of apnoeas per patient	2.5 (2–5.75)
Number of apnoea episodes where an RRp could be calculated [n (%) of total detected apnoea episodes]	54 (81)
Number of apnoea episodes resulting in hypoxaemia [n (%)]	14 (21)
Elapsed time until hypoxaemic episode(s)	40.3 (29.0–94.0)

Data represent median with interquartile range or frequencies. Apnoea = RRc 0 brpm for >36 s. Hypoxaemia = SpO_2_ < 92 %

*RRc* respiratory rate for capnography, *RRp* respiratory rate for plethysmography

### Level of agreement between RRc and RRp

**Fig. 2:**
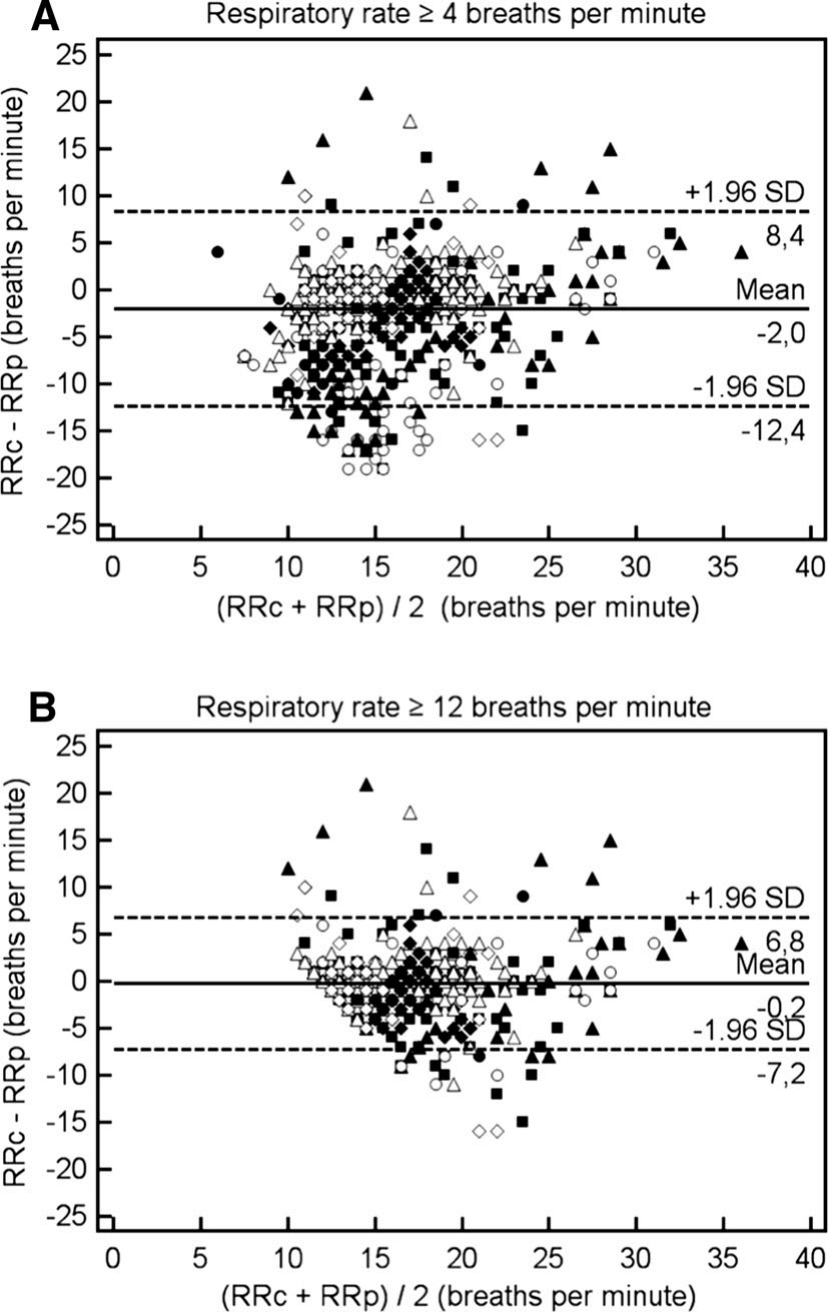
Bland-Altman analysis corrected for repeated measures of capnography respiratory rate (RRc) versus plethysmography respiratory rate (RRp) in case of an RR ≥ 4 breaths per minute (brpm) (**a**) or an RR ≥ 12 brpm (**b**)

The photoplethysmography device did not report an RRp in 15.5 % of all recorded minutes. The level of agreement between the RRc and RRp was evaluated using Bland–Altman (BA) analysis corrected for repeated measurements in case of a minimal RR of ≥4 brpm (Fig. 2, panel a) of RR of ≥12 brpm (panel b). Panel a shows in 690 paired observations of RR data a bias of 2.25 brpm (SD 5.41), with 95 % limits of agreement from −8.35 to 12.84 brpm.

**Fig. 3:**
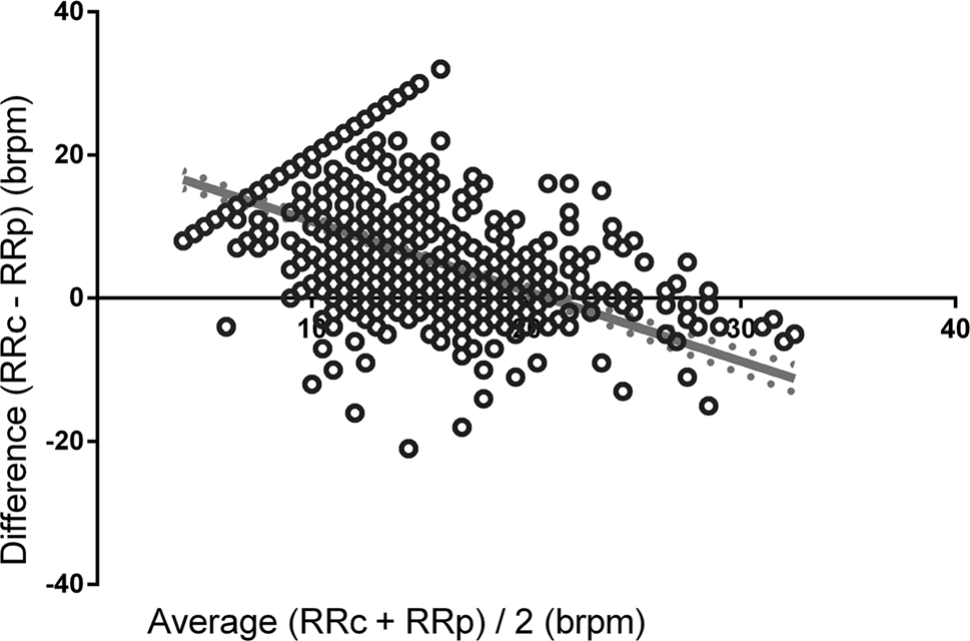
*Linear* regression analysis using (RRc + RRp)/2 as independent variable and RRc–RRp as the dependent variable represented as separate *dots*

Linear regression analysis revealed a slope of −1.026 (*P* < 0.0001) for the relation between the difference between RRc and RRp for every mean RR, suggesting that the difference between RR measurements decreases as the average respiratory rate increases, until a point where this difference becomes negative (Fig. 3). Using a cut-off of an RR ≥ 12 brpm, 495 paired observations revealed a bias of 0.50 brpm (SD 3.18) with 95 % limits of agreement from −5.72 to 6.73 brpm (Fig. 2 panel b).

## Discussion

The present study investigated the level of agreement between the respiratory rate as calculated from the respiratory variation during photoplethysmography with capnography as gold standard during procedural sedation and analgesia for upper gastrointestinal endoscopic procedures. Our study showed a low level of agreement between photoplethysmography and capnography respiratory rate (RR), even when the analysis was limited to normal breathing frequencies (RR ≥ 12 brpm).

Respiratory rate monitoring might be used for the early detection of respiratory depression and prevention of hypoxaemic episodes. We therefore studied the added value of photoplethysmography respiratory rate to capnography in the detection of hypoxaemia during sedation. As the photoplethysmography device is unable to detect apnoea, we evaluated the occurrence of bradypnoea before a hypoxaemic event, and found a low incidence rate for both devices. Our findings suggest that the addition of a photoplethysmography respiratory rate to standard capnography in procedural sedation does not increase the chance to detect apnoea or bradypnoea before the occurrence of hypoxaemia. The clinical implications of our findings should however be further tested in a randomized controlled setting.

In contrast to our findings, Addison and colleagues recently reported a good level of agreement between the RR as measured by plethysmography and capnography [13]. One explanation for these different findings is the removal of capnography waveforms with poor quality in their study, while we choose to keep all capnography recordings in the final analysis to mimic routine clinical practice. Moreover, in contrast to other studies we included patients with comorbidities and high median ASA scores [1, 16], and consequently reported a relatively high number of hypoxaemic episodes. Age, male sex, comorbidities, fentanyl use, ASA classification, and BMI were previously reported as independent risk factors for respiratory events during UGI endoscopy [1, 2, 16, 17]. In contrast, Goudra et al. [18] found no increased risk for hypoxaemia in patients with a higher ASA classification or BMI when patients were pre-oxygenated with 100 % oxygen prior to drug administration and received an airway device to maintain a patent airway. Our study shows that a hypoxaemic episode was preceded by apnoea in only in 34 % of the cases, with a delay of about 40 s between apnoea and the development of hypoxaemia. This delay was comparable as reported by van Loon et al. [19]. However, in the study of van Loon et al. [19], supplemental oxygen was only provided in case of disturbed oxygenation or ventilation, which makes these findings incomparable with our report.

As the depth of sedation is of influence on the occurrence of hypoxaemia, we compared anaesthesia infusion rates with other studies. Unfortunately, most studies lack information with respect to anaesthesia infusion rates, which makes a close comparison with our findings unfeasible. A limitation of our study was that the degree of sedation could not be objectified with bispectral index spectrometry (BIS) because s-ketamine would have reduced its value in predicting moderate sedation levels [20].

Although capnography is recommended in the guidelines during PSA for UGI endoscopy, it may be difficult to use capnography as a diagnostic tool by itself, and it should be used in conjunction with accompanying data, such as heart rate, blood pressure and photoplethysmography. The value of capnographic recordings might be enhanced if the shape of the capnogram, the P_ETCO2_, and respiratory rate are added to algorithms for clinical decision-making [21]. Deitch et al. [22] reported 100 % sensitivity for hypoxia detection with capnography while supplemental oxygen was administered. Respiratory depression was set as a P_ETCO2_ of >50 mmHg, an absolute increase or decrease from baseline P_ETCO2_ of 10 % or greater, or loss of the CO_2_ waveform for >15 s. In this study, 50 % of respiratory depression events were caused by an P_ETCO2_ > 10 % below baseline, suggesting that the prognostic value of capnography might be enhanced by evaluation of more advanced indices. However, extrapolation of these indices to the setting of UGI endoscopy might be difficult, as the endoscope disables airflow in a narrowed airway, especially in case of obesity, and PSA-related decreases in cardiac output may influence the P_ETCO2_. Moreover, as we studied the routine clinical setting instead of a controlled setting where a dedicated healthcare provider could act on disturbed capnographic values [8, 22], our data are difficult to compare with other reports.

Our data showed that the Nellcor 2.0 was able to register the respiratory rate in 81 % of the apnoea episodes detected by capnography. However, the Nellcor did not improve hypoxaemia prediction as surrogate end-point of patient outcome, when compared to capnography. The analysis of respiration-induced variations in the plethysmography waveform requires frequency analysis of the PPG baseline [23]. As breathing movements disappear during central apnoea, the phasic PPG respiratory signal vanishes, while the PPG signal can additionally be influenced by autonomic nerve activity [24]. Nilsson et al. [25] additionally compared different PPG techniques at different anatomical sites for synchronous measurement of heart rate, respiratory rate and oxygen saturation, and showed that the location of the finger is the least favourable for RR detection. Although there may be a timing deviation between different sensor location, this deviation was considered as clinically insignificant in the present study. Moreover, the largest respiration-induced variations in the PPG signal were observed at high tidal volumes, absence of abdominal breathing and low respiratory rates [24]. During propofol-induced procedural sedation, tidal volume is probably more suppressed than respiration rate [26–28]. Although abdominal respiration is diminished during spontaneous breathing in sedation [29], we showed that the difference between RRc and RRp changes with distinct respiration rates. A more accurate RRp due to the increase in respiratory effort and thoracic expansion with high inspiration rates might explain this [30].

Our study is pragmatic by nature, and we aimed to include all measured waveforms in our final analysis in order to allow conclusions that are relevant to daily clinical practice. Although this might have contributed to a lower level of agreement between methods, our final conclusions are better implementable in the clinical setting.

The question that arises from the current study is whether capnography as gold standard for respiration rate during UGI with procedural sedation is the most appropriate methodology as comparator. While the Nellcor device was unable to detect low respiration rates, the capnogram was frequently disturbed by the endoscope and alterations in airflow. Additionally, Holley et al. [10] recently performed a study with the ExSpiron bio-impedance-based respirator volume monitor, showing that low RR values do not represent episodes of respiratory depression, and the association of respiration rates with minute ventilation in upper endoscopic procedures was very low. This manufacturer-supported trial included 51 patients to simulate a variety of RR alarm conditions. The study showed that a substantial fraction of low minute ventilation (MV) measurements (MV < 40 % of MV baseline) went undetected at 8 brpm (>70 % low MV measurements were missed), but no hypoxemic event analysis was performed. In addition, Ebert et al. [31] described a −48 % reduction of minute ventilation by bioimpedance following sedation. Future studies should investigate whether other measurements, like respiratory minute volume, are superior to detect respiratory compromise and prevent hypoxemia in sedated patients when compared to available techniques, such as respiration rate monitoring or capnography.
